# Immune system activation through immunogenic cell death and tumor recruitment of dendritic cells is required for anti-tumor activity of a plant-derived polyphenol rich fraction

**DOI:** 10.1186/2051-1426-3-S2-P301

**Published:** 2015-11-04

**Authors:** Alejandra Gomez-Cadena, Amaia Martinez-Usatorre, Claudia Urueña, Karol Prieto, Alfonso Barreto, Pedro Romero, Susana Fiorentino

**Affiliations:** 1 Grupo de Inmunobiología y Biología Celular. Pontificia Universidad Javeriana, Bogotá - Colombia, Bogota, Colombia; 2 Ludwig Cancer Research Center, University of Lausanne, Lausanne – Switzerland, Epalinges, Switzerland

## 

Chemotherapy faces the problem of chemo-resistance in large part of tumors, leading to the appearance of metastasis difficult to eliminate. Over the past decade an improved understanding of the effect of immunogenic chemotherapy on tumor-host interactions has led to the concept of immunogenic cell death (ICD), a type of cell death capable to convert a tumor into an *in situ* vaccine through the generation of danger signals, dendritic cells activation an finally breaking tolerance, which leads to the destruction of residual tumor cells. Polyphenols are natural compounds from plants, traditionally used for cancer treatment, that exhibit multiple biological activities including anti-oxidation, anti-angiogenesis and pro-apoptosis, which together may exert potent anti-tumor activity. However, little is known about the mechanism of action of polyphenols. We previously obtained a normalized polyphenol rich fraction from *Ceasalpina spinosa* (P2Et) that exerted cytotoxic activity on several tumor cell lines. Moreover, P2Et showed anti-tumor activity in the 4T1 transplantable model through ICD induction. Vaccination with P2Et-pretreated 4T1 cells yielded long lasting *ex vivo* IL-2, TNF-α, IL-4, IL-5, and IFN-γ secreting multifunctional T cells after specific 4T1 cell stimulation. In this work we investigate the activation of the immune system after *in vivo* P2Et treatment of B16 tumor bearing mice. Analysis of spleen, lymph nodes and tumors shows increase numbers of CD44 high, CD4 and CD8 T cells, as well as NK cells in P2Et treated mice compared to non-treated ones. In addition, *in vitro*, phagocytosis of P2Et treated tumor cells induce activation of DC, and *in vivo* P2Et induces recruitment and activation of cross-presenting DCs (CD11c^+^, CD11b^+^, Ly6C^+^ or CD11c^+^ CD8α^+^) in spleen and tumors, which could lead to the effective activation of CD8 T cells favoring a better tumor control. In fact, vaccinated mice treated with P2Et cells are able to effectively control the growth of transplanted tumor. Accordingly, we observed that P2Et anti-tumor activity is highly dependent on an intact immune system, as P2Et anti-tumor activity is lost in RAG γc KO immunodeficient mice. In summary, P2Et exert its anti-tumor activity through the activation of the endogenous immune system, playing an important role not only in the destruction of the primary tumor but also in controlling metastasis.

